# Maturation Differentially Regulates the Pharmacological Interaction Between Protein Kinase C and BK Channel Activity in Ovine Middle Cerebral Artery

**DOI:** 10.64898/2025.12.03.692156

**Published:** 2026-06-05

**Authors:** Michell Goyal, Ravi Goyal

**Affiliations:** 1 Department of Physiology, University of Arizona, Tucson, AZ 85724; 2 Department of Obstetrics and Gynecology, University of Arizona, Tucson, AZ, 85724

The authors have withdrawn their manuscript because they want to incorporate additional data and further analysis. Therefore, the authors do not wish this work to be cited as reference for the project. If you have any questions, please contact the corresponding author.

